# Qigong Exercise and Arthritis

**DOI:** 10.3390/medicines4040071

**Published:** 2017-09-27

**Authors:** Ray Marks

**Affiliations:** 1 Department of Health and Behavior Studies, Program in Health Education, Columbia University, Teachers College, New York, NY 10027, USA; rm226@columbia.edu; Tel.: +1-212-678-3445; Fax: 1-212-678-8259; 2 School of Health and Professional Studies, Department of Health, Physical Education & Gerontological Studies and Services, City University of New York, York College, Jamaica, NY 11451, USA

**Keywords:** arthritis, exercise, quality of life, Qigong, rehabilitation

## Abstract

**Background:** Arthritis is a chronic condition resulting in considerable disability, particularly in later life. **Aims:** The first aim of this review was to summarize and synthesize the research base concerning the use of Qigong exercises as a possible adjunctive strategy for promoting well-being among adults with arthritis. A second was to provide related intervention directives for health professionals working or who are likely to work with this population in the future. **Methods:** Material specifically focusing on examining the nature of Qigong for minimizing arthritis disability, pain and dependence and for improving life quality was sought. **Results:** Collectively, despite almost no attention to this topic, available data reveal that while more research is indicated, Qigong exercises—practiced widely in China for many centuries as an exercise form, mind-body and relaxation technique—may be very useful as an intervention strategy for adults with different forms of painful disabling arthritis. **Conclusion:** Health professionals working with people who have chronic arthritis can safely recommend these exercises to most adults with this condition with the expectation they will heighten the life quality of the individual, while reducing pain and depression in adults with this condition.

## 1. Introduction

Arthritis, a generic term describing over 100 different forms of joint disease, consistently produces varying degrees of pain, stiffness, and swelling in or around one or more joints, regardless of arthritis type. As a result, when symptomatic, arthritis can be extremely debilitating, and is often associated with a decreasing ability to function physically, a poor or limited aerobic capacity and exercise endurance, as well as excess fatigue and sleep problems. Some forms of arthritis, such as rheumatoid arthritis and lupus, can also affect multiple organs causing widespread symptoms of ill health. As in other chronically painful disabling conditions, arthritis can have a very negative effect on a person’s mental health [1] and can severely impair an individual's ability to carry out his/her normal functions of daily living without compromise, effort and excessive physical stress [2].

Unfortunately, despite the considerable disability associated with arthritis, no cure for any form of this condition prevails. In addition, while useful in restoring function and ameliorating pain in severe cases of the disease, not all forms of arthritis are amenable to artificial joint-replacement surgery. As well, efforts to reduce pain accompanying the disease by means of various analgesics, injections, and/or nonsteroidal anti-inflammatory drugs, which constitute the most common non-surgical approaches to treating arthritis, frequently prove ineffective, or produce adverse systemic effects causing the articular cartilage lining the joint to disintegrate. Further, rest does not bring as many benefits as once expected, and can increase debility and joint destruction processes. Finally, since no drug reverses or delays the progression of arthritis, and some drugs used to minimize pain are addictive, their long-term efficacy has been challenged. As a result, adjunctive methods that might reduce the pain and disability associated with arthritis (other than medications or surgery), have been advocated for some time [3].

In this regard, physical activity is often recommended for purposes of promoting general health and well-being, as well as cardiorespiratory function, and exercise endurance among people with arthritis [4]. Further health benefits derived from physical activity participation include assistance in weight reduction [5], improvements in joint flexibility and muscle strength [6], decreases in the severity of anxiety and depression found among people with arthritis [7], as well as improving their mood and sense of well-being [8].

However, when even the shortest bout of exercise may cause pain and leave the arthritic patient with more, rather than less pain [6], and the thought of pursuing any form of exercise may be strong enough in its own right to deter participation in this important treatment form, an activity approach that is both efficacious as well as nonstressful and that can be adhered to in the long-term is strongly indicated. As well, exercises recommended for people with arthritis must be safe biomechanically speaking, because their affected joints are commonly very vulnerable to undue mechanical insults and stressors. Indeed, while exercise is touted as a ‘cure-all’ for almost all chronic health conditions, exercises that overexert this patient group or cause excess impact to fall on their already damaged joints are strongly contra-indicated. In particular, excessive joint loading as a result of exercise can increase joint inflammation that produces pain, along with an aversion to exercising and a preference for being sedentary, muscle weakness, and contractures, and poor cardiovascular endurance. Thus, noninjurious exercise approaches, especially those that can improve life quality, reduce pain, and depression, while safely heightening the functional capacity of people with arthritis, are strongly desired and highly indicated.

Indeed, based on a growing literature of failures in treating any form of arthritis, and successes in treating various chronic diseases other than arthritis as well as most of the symptoms associated with arthritis with Qigong exercise, it appears a strong case can be made to support its application as an adjunct or standalone intervention for people with arthritis, although almost no substantive research in this realm currently exists to support this view.

This narrative review thus examines the results of related research concerning the possible impact (what can occur after Qigong exposure), efficacy (whether Qigong shows promise relative to standard practices under controlled conditions) and effectiveness (whether Qigong practice produces favorable outcomes in real world situations) of Qigong exercise for people with arthritis. It specifically examines whether Qigong is a potentially safe and efficacious exercise approach for advancing the well-being of people with arthritis who commonly have incurable multiple overlapping physical and mental health challenges and very fragile painful joint structures that can be made worse by incorrect exercise applications or failure to exercise (See Table 1).

It was hoped that despite the lack of any systematic body of research in the context of arthritis that this narrative and descriptive overview of Qigong exercise would provide a strong case for considering its utility in arthritis care approaches. In addition, this paper will provide some tentative recommendations for improving the well-being of the many people worldwide who suffer from disabling arthritis, including children. It was especially hoped the review would sensitize health practitioners concerning the potential benefits of Qigong for their arthritis patients, while expecting this strategy to effectively heighten their physical and psychological well-being in a safe manner. It was also hoped that researchers could possibly examine this data and consider how they might make contributions of long-lasting impact to the field by extending the preliminary efforts described in this review in the context of this most challenging health realm of arthritis care and arthritis rehabilitation.

## 2. Methods

To obtain the desired data, the electronic data sources Academic Search Complete, PubMed, Scopus. Google Scholar, and Web of Science were searched. The search years ranged from January 2007–August 2017 and the key words included the terms *“arthritis”* and *“Qigong”*. Excluded were non-English based articles without English abstracts. Because only a limited number of empirical studies related to the present topic were found, a review of studies pertaining to the use of Qigong for common symptoms of arthritis, rather than any specific form of arthritic disease was implemented. The results of these studies are discussed in this report largely in relation to their possible application for treating one or more arthritis symptoms, regardless of type of arthritis. All forms of study design were deemed acceptable, and the value of this meditative movement form was assessed directly where possible, as well as in instances where it was deemed the application might be helpful in the context of arthritis management. Although tai chi quan can be practiced as Qigong, studies focusing solely on tai chi, but that did not mention Qigong specifically, were excluded [9]. Otherwise, all definitions of Qigong, and forms of Qigong applied to adults with some form of arthritis and/or arthritis like symptoms were deemed acceptable and are described in narrative form because it is impossible to consider subjecting such limited and highly diverse data of heterogeneous samples and methodologies to any form of meta-analysis.

### Search Results

A total of 75 generally relevant papers or articles were retrieved. Of these, only a small number of studies were directly related to arthritic conditions. For example there were only 16 listed studies in PubMed of the 538 studies on Qigong, and the populations studied were diverse, including rheumatoid arthritis, fibromyalgia syndrome, osteoarthritis, and ankylosing spondylitis, and most were observational, case, or pilot studies. There were many more publications related to symptoms found in arthritis such as pain, anxiety, depression, and low self-efficacy, but these did not focus on arthritis. Other studies focused on cancer and Qigong, type 1 diabetes, animal models and Qigong effects, group Qigong for anorexia, Parkinson’s Disease, heart disease, and respiratory problems, among others, and were not appropriate for this specific review. However, even though markedly limited in number and quality, with inconsistent post-treatment effects, some preliminary comparative studies have shown Qigong to be potentially as effective as standard treatment and possibly less stressful on joints than standard exercises. In addition, despite the small number of conducted studies, and the diverse samples examined and exposed to many different forms of Qigong, some showed long-term benefits even in highly challenging arthritis conditions. As well, even though the mode of Qigong applied was not commonly grounded in any set of physiological principles, and no two studies applied the same strategy, promising results were more common than not. Moreover, even though some studies tested the use of external Qigong involving massage, touch, and therapist-derived focused attention and others examined internal Qigong involving self-practice [9], some success was evident with both approaches.

In sum, assuming very few studies have been omitted from this very extensive search, if we were to limit and report only on studies directly related to the topic of examining the utility of Qigong for arthritis, and to require the paucity of available studies to have rigorous designs, no chapter or review of the topic could be undertaken at this present time.

To provide the reader with a context for examining this topic in the future, the next section highlights some studies that reflect the potential for Qigong to be used effectively as an efficacious therapeutic intervention in general, and for arthritis symptoms more specifically. No prior publication on this current theme could be found, so it is believed this chapter can serve as a first step in systematizing this line of inquiry, bearing in mind the need for caution in interpreting this body of information.

## 3. Qigong as Exercise Therapy

Qigong and its many variants have been used to promote health for more than 5000 years [10]. The approach, which is an ancient component of traditional Chinese medicine [11], predominantly involves breathing exercises [12], meditation, and gentle slow body movements [11] and is a system of yoga designed to reduce tension and quiet the mind [13]. Practicing Qigong in one of its numerous forms, which can be subclassified as spiritual, healing, medical, or martial Qigong [9], has been shown to help people overcome general or specific health problems that result in physical limitations and disability as well as psychological distress. Based on the idea that the flow of energy through the body can influence health status, Qigong movements are energy exercises [14], which may reduce sympathetic nervous system stimulation, and inefficiencies in upper thoracic breathing as well as anxiety, while improving peace of mind and life quality [13]. These exercises can be applied in sitting, walking, standing, or even when reclining with few side effects and can be practiced by the individual or through the mediation of an external practitioner [14]. These non-strenuous slow-motion exercise movements, as well as mindful concentration and controlled breathing designed to heighten energy flow or qi [14], appear to helpful even if only practiced 15 min per day [11].

Aimed at fostering physical, psychological and spiritual well-being [11], the specific body movements advocated in Qigong, along with having a relaxed state of mind [11] may be especially useful for the arthritis sufferer even if they are confined to their beds or have limited physical capacity. The skills involved in practicing Qigong that can be commonly acquired through regular practice, are also recognized for their other broad based therapeutic health-related benefits, including those that involve the immune system [11] and sleep quality [11,15], balance and agility [16], and interest in life [15]. While the term Internal Qigong refers to the self-practice of breathing, and mind-body techniques, External Qigong consists of similar precepts but is received passively, through a skilled practitioner rather than the client [9], and involves hand movements, focused attention, and mind healing techniques designed to direct the energy or ‘qi’ of the therapist to the patient or client [17].

Although very few arthritis-related studies have been conducted, Rogers et al., [10] who reviewed clinical trials of Qigong as applied to older adults, found overall potential benefits in physical activity, and falls risk reduction [18]. Qigong participation can also reduce prevailing levels of anxiety and high blood pressure [19]. As outlined in Table 1, these are all problems often associated individually or collectively with the different forms of arthritis. Other benefits may include improved attention, brain processing, and overall capacity [19], reductions in required medications, greater health benefits than medication alone, an improved ability to relax, and more flexibility of affected joints, plus more resistance to disease [9]. As well, Qigong participation may promote healing [12], and improve flexibility, while reducing pain [20]. Other data indicate Qigong practice can help to favorably alter lower extremity alignment, as well as posture and balance [13], and may yield important metabolic and psychological effects if combined with tai chi [21]. Qigong is also found beneficial for fostering life quality, sleep quality [22], hand grip strength, and trunk mobility [23].

Moreover, a limited number of cardiovascular risk factors, such as high blood pressure [10,24], abnormal lipid profiles [18] and heart rate irregularities [23], often experienced by people with arthritis, may be positively influenced by Qigong exercise participation. In addition, Qigong exercises have been found to enhance the participant’s perceptions of self-efficacy, an improvement health determinant that could impact an arthritis patient highly positively and significantly in its own right. Improvements in physical as well as emotional well-being [10,16] and pain, plus a strengthened immune system may similarly result by consistently practicing this exercise form of gentle movements, breathing techniques, meditation, and self-massage [25].

In addition, Hou et al. [26] found moderate quality evidence as regards the effects of Qigong on physical function in patients with condition known as osteoarthritis. The authors who examined different forms of Chinese medicine concluded Qigong was an effective method for improving function in this patient population. It was further suggested that Qigong practice may specifically help arthritics who suffer from excess muscle tension that can cause pain and possible excess joint compression, as well as fostering a state of reduced anxiety and depression, while heightening peace of mind and life quality [13]. If applied carefully, Qigong exercises can be directed towards altering the direction of muscle pull at selected joints, which is often problematic, thus helping to alleviate joint stress, and potentially enabling more effective muscle function and mobility [13]. The nature of Qigong exercises may also help to heighten adherence to recommendations to perform exercises regularly among this patient group, especially if the instructor is engaging and compassionate, and raises expectations for overall well-being [13].

Chen et al. [27], who conducted a pilot trial of Qigong therapy for 10 cases with arthritis, found improvements in pain and movement disability was decreased as was anxiety for a considerable period of post-Qigong treatment. Also noteworthy is the fact that Qigong participation can benefit body composition positively [16,28], which could be extremely helpful for people with arthritis, who are often excessively overweight. However, according to results of a recent randomized controlled trial that examined the effects of external Qigong therapy for people with knee osteoarthritis, if this form of Qigong is used, the benefits may vary dependent on the characteristics of the healer [27].

The type of Qigong approach may also play a role in its effectiveness. An et al. [29] noted Baduanjin-style Qigong resulted in improvements in muscle strength, knee pain, stiffness and function during an 8-week training period. No control group was studied, but the short-term improvements in aerobic capacity with no adverse effects were notable. Moreover, in a controlled study of long-term care residents, a positive effect of Laughing Qigong was evidenced in terms of cognitive impairments and depression [30], and these were accompanied by increases in cortisol levels.

In short, Qigong is a method of gentle self-healing exercises [25] designed to increase well-being. It can help to strengthen the body’s musculoskeletal system and cognitive function [19], increase joint flexibility, motor function, and movement coordination. It can also help activate postural improvements, and diminish pain. In addition, its application can reduce fatigue [22], stimulate the circulation, correct systemic imbalances [25], and optimize the body’s physiology. It can be practiced by all age groups without equipment in either standing or sitting or reclining. Accordingly, it is unsurprising that Hou et al. [26] who conducted a systematic review of randomized controlled trials of traditional Chinese medicine for osteoarthritis, found Qigong applications that combined motion effective for treating this type of chronic illness and its severe impact on physical function and well-being. See Table 2 for examples of randomized studies.

## 4. Additional Research Evidence

As outlined above, several lines of evidence from a variety of studies support the potential efficacy of Qigong as a helpful and safe form of exercise for people with arthritis, especially its psychophysical health symptoms [38].

Manzaneque et al. [39] for example, found improvements in psychological health status and sleep duration after one month of practice. The therapy was conducted in groups three times per week, with the addition of some individual exercises. The method used was termed ‘the eight pieces of silk brocade’ that incorporates only one set of movements, practiced in succession. The sequence was repeated eight times and contained eight distinctive movements and lasted for 30 min. It was guided by a qualified instructor, but subjects were encouraged to carry out the movements on their own. Control subjects followed their usual daily routines. Differences between the groups were estimated by subtracting differences in pre-test scores of desired variables. Since people with arthritis commonly suffer from anxiety, depression, and sleep disturbances, the findings of this study were very promising. It is possible too that a study of longer duration would have revealed immunological improvements as well, which are also very important in the context of some forms of arthritis. In favor of this idea, as outlined in Table 2. Vera et al. [11] found positive immune responses after one month among an experimental group of subjects, but not among the control group after subjects practiced Taoist Qigong. In this randomized study, experimental subjects carried out these exercises on a daily basis if possible for one month, whereas control subjects performed their usual routines. Each movement carried out by the experimental subjects was repeated six times and involved a focus on breathing. An instructor guided the practice formally three times a week in a group.

In another study, Lee et al. [40] who conducted pilot work in the community to examine the effects of tai chi Qigong for purposes of improving life quality in 44 elderly people with knee osteoarthritis, found that the eight-week program of 60 min sessions twice weekly yielded greater improvements in life quality when compared to wait list controls. Walking ability was also significantly improved as outlined below in Table 3:

However, unlike Chen et al. [17], who found a significant reduction in scores for assessing knee osteoarthritis disability called the WOMAC after applications of Qigong therapy applied externally to 112 adults, Lee et al. found no similar improvements differences between their active or control subjects. Chen et al. also noted that different healers produced different pain and functional outcomes. Benefits lasted up to three months regardless of group allocation.

As well, Tsai et al. [41] found Qigong improved the physical status including muscle endurance, and waist-to-hip ratio and body fat of participants. Here the method used was described as the Muscle/Tendon Change Classic, which is characterized by simple, slow, and full body exercises that seemed to help the experimental group to show health preserving effects after 8 weeks of training. Sakata et al. [42] similarly, found a 12-week Qigong exercise program to increase walking speed, along with reductions in body fat, an approach that could be highly beneficial for many people with arthritis. The improved use of vestibular input and wider stances by which Taiji-Qigong may not only improve balance in older adults [43], but may also be very helpful for arthritis sufferers who have balance deficits as well as for those who are prone to falls, without any safety concerns that might arise with other forms of exercise [29].

Among other noteworthy benefits of Qigong as applied to the arthritis patient are long-lasting improvements in sleep health [31], as well as reductions in fatigue in those with chronic fatigue [32], a common arthritis symptom, plus chronic pain [44]. Although von Trott et al. [45] found Qigong produced benefits commensurate with traditional exercises among older adults with chronic neck pain and no benefits over the control condition, it was also shown that more of these elderly subjects examined chose to continue Qigong rather than regular exercise when given the choice. Coleman [46] similarly reported that Spring Forest Qigong and its active exercise and meditation components were effective self-care approaches for persons with chronic pain. While Bai et al. [47] did not study arthritis patients, their meta-analysis of 10 studies showed internal Qigong generates benefits that exceed those of control subjects.

These findings are all highly relevant to the treatment of intractable arthritis problems, and even if more study is desirable, and it is impossible to say what form of Qigong is best, the improvements in arthritis symptoms in available studies post Qigong therapy do appear to be worthy of attention. Moreover, despite the lack of rigor in the reported studies, the benefits that accrue do appear to be based on a set of sound physiological principles and general study findings. These include, but are not limited to the ability of Qigong practice to foster muscle relaxation, blood flow and delivery of nutrients, as well as pain-killers and other drugs, plus the more efficient removal of pain mediators [17]. Better aerobic capacity [29], reductions in anxiety and the anti-depressive effect of Qigong [38,48] are further explanations as to why Qigong may help to reduce arthritis pain symptoms. Its use as a tool for ‘spiritual exploration’, its observed effect on decreasing in blood pressure and improving function among older adults are also highly relevant [10] [See Box 1].

Box 1 **Specific Outcomes of Qigong that Could Promote Well-being Among People with Arthritis. Adapted from: Rogers et al. [10]; Gallagher [13]; Vincent et al. [14]; Coleman et al. [15]; Liu et al. [21]; Craske et al. [22]; Lee et al. [24]; Posadzi [49]; Manzeque et al. [39]; Yang et al. [43]; Lynch [31]; Teut et al. [46]; Liu et al. [48]; Benhamon et al. [50]; Wang et al. [51]; Goldrosen et al. [52]; Lauche et al. [53]; Mannerkorpi and Arndorw [54]; Singh et al. [55]; Chang et al. [56]; Sancier et al. [57]; Ho et al. [58].**

■ Increased aerobic capacity and energy
■ Improved balance
■ Improved blood pressure control
■ Improved fitness
■ Improved flexibility
■ Improved functional ability
■ Improved general health status
■ Improved immune function
■ Improved metabolic health
■ Improved mental attitude and function
■ Improved mood state
■ Improved movement harmony
■ Improved muscle strength
■ Improved muscle tone
■ Improved sleep health

## 5. Discussion

Although modern medicine has been successful in managing infection and saving the lives of trauma victims [6], preventing or treating the extent of the disability associated with arthritis remains extremely challenging. Since pharmacologic and surgical approaches are often limited or contra-indicated in ameliorating arthritis in it various forms, a growing evidence base suggests alternative low risk approaches such as Qigong—an ancient form of Chinese meditation breathing exercise [59] and therapy [60]—might be highly advantageous in promoting mobility and participation in physical activity among people with arthritis, even those with an advanced stage of the disease. Moreover, adherence to exercise, a key health recommendation for this group, may be heightened because Qigong offers a low intensity exercise approach based on tai chi [60] including slow, graceful, low impact, and low velocity movements that do not involve a lot of bending or joint stress. These movements can indeed promote both physiological and psychological health [25], including posture, coherent breathing [61], and sleep duration [39], while reducing negative affective states such as depressive symptoms [48], and can be verified scientifically to be accompanied by objective changes in cerebral brain activity [62], as well as by other objective tests. Importantly, Qigong, a diverse set of exercises including breathing exercises and self-massage, also enhances muscle function, as well as physical and functional capacity plus overall well-being even when only practiced for short periods of time. Moreover, it is said that the exercise components and goals of Qigong are possibly well aligned for treating arthritis if it can be shown that this disease is attributable to blocked or unbalanced qi [63], and can be adapted for frail elders where movements are too difficult [38].

Research shows Qigong is also cost effective, safe to implement, can help to reduce medication dosages [12], improve bodily experiences of older persons with chronic pain [64], and provide for better health benefits than medication alone [12]. Requiring no equipment, it is easy to learn [29]. As well, the exercises produce effects commensurate to oftentimes time consuming education therapies [65], can be carried out independently in diverse postural positions, and can hence be advocated for most people with arthritis, regardless of age or extent of disability or language barriers. Additionally, this form of therapy appears to be sufficiently flexible to accommodate different people’s preferences for exercise quite successfully, given its many different approaches.

Qigong is associated with an exercise intensity that may be less injurious to joints than commonplace high intensity exercises often advocated for improving aerobic capacity. As such, Qigong may provide a safe form of exercise for reducing chronic fatigue [22], as well as stress, while promoting important physical and mental health benefits that are likely to foster an arthritic patient’s independence and well-being quite significantly. Additionally, even if the disabled individual cannot exercise actively, it appears external Qigong can be applied to promote muscle relaxation and blood flow in an effort to reduce their pain [63].

In addition, people with arthritis who are generally reluctant to adhere to activity programs necessary to keep them mobile and functional, may face fewer exercise challenges when considering Qigong because Qigong is generally a pleasurable series of nonstressful activities for most people. Despite the fact that Qigong applications are not totally successful when examined in the context of some meta-analyses e.g., [66], the fact that Qigong might be helpful in encouraging and motivating people with arthritis to not only participate in, but to adhere exercise participation over the long-term, and results indicate Qigong may be an especially viable option for patients with unstable joint problems or osteoporotic joints, this ancient set of meditative exercises may prove highly valuable for fostering the well-being of this prevalent patient population. This is especially so because adults with any form of arthritis who fail to participate in consistent exercise activities, commonly incur worse joint pain, sleep challenges, depression, and dysfunction. They may hence be more challenged economically, socially, and functionally than they need to be.

However, to overcome the possible issue of placebo effects [22], publication bias [48], considerable anecdotal rather than statistical evidence, plus the dearth of specific short-term and long-term follow-up studies, studies on specific well-defined samples and adequately powered studies, more attention to overcoming the numerous design issues that prevail in available studies [53,54,66], along with adequate control interventions [64] are clearly indicated before any firm conclusions can be derived about this promising modality. In addition, to more ably demonstrate the benefits of Qigong for improving the various arthritis related challenges such as poor balance control, posture, heightened distress, joint stiffness, depression, sleep problems, and pain, more careful, high-quality research with well-defined research questions, inclusion criteria, robust research designs, Qigong applications with well-defined parameters, and assessments using validated outcome measures is clearly indicated. Comparative studies of various Qigong approaches, dosages, and modes of delivery are also greatly indicated for guiding clinical practices.

## 6. Conclusions

Although the literature is limited in depth and its validity can be questioned, in light of the magnitude of the immense public health burden associated with arthritis and the likelihood of this increasing—rather than decreasing as society ages—and the finding of no short-term detrimental effects post-Qigong participation on arthritis disease activity, the potential for Qigong to enhance the well-being of these sufferers should be explored further. To this end, well-designed studies of separate forms of arthritis, and methods of applying Qigong will be helpful. Studies that examine the interesting mechanisms underlying the observed benefits of Qigong, for example the possible influence the modality might have on the nature of the participant’s electromagnetic energy and nerve transmission processes [49], may also be highly revealing. What forms of Qigong yield optimal results, and whether Qigong exercises can stimulate bone growth and connective tissue repair among people with arthritis, would also be of great interest to examine, as would its impact on the immune system, falls risk, and cognitive function.

In the meantime, arthritis remains the most prevalent chronic disease disabler, and to assist people with one or more of these debilitating conditions to acquire a better life quality, notwithstanding the limited quality of the prevailing scientific basis for this, it appears clinicians should not overlook the potential wide-reaching physical and psychological benefits of Qigong for people with arthritis. Indeed, Qigong participation would be expected to provide a fair percentage of arthritis cases significant relief from the painful symptoms of arthritis without the damaging side effects of many other forms of intervention [67], and may be reasonably commensurate in impact with more standard approaches as suggested by Blodt et al. [68] and Holmberg et al. [64]. The fact that the movements involved aim to protect internal organs from harm, and are performed more slowly than more Westernized exercises, and involve thinking and concentration, rather than mindlessness, may however provide a unique complimentary approach to reducing both fatigue and pain, as well as depression and anxiety [22,47,50,57,61]. They may also help participants to deal more effectively with mental as well as physical stressors without the use of dangerous drugs [17], and permit arthritis patients to be active rather than passive partners in their recovery. As well, Qigong appears to foster better postural control, flexibility and balance that may help to prevent falls that can lead to further disability and disablement. Qigong participants also appear to have better sleep quality [69], immune system functioning [69], blood glucose levels [70], and blood pressure ratings [23].

Taken as a whole, and notwithstanding the need for more definitive research in this realm, it does seem clinicians can still be encouraged to explore how Qigong might be helpful for their clients with arthritis, as based on the available data, along with the attributes of arthritis and the immense need for alternative approaches to fostering a high life-quality for this large group of patients. In particular, as suggested by findings of Chang et al. [71], they can possibly help their clients to locate professional or expert Qigong instructors, and sites in the community where they can undergo assessments, training and follow-up with the expectation they will possibly benefit in one more arthritis-associated realms. Specifically targeted for the practice of Qigong might be arthritis patients with comorbid diseases such as heart disease and/or diabetes, those who are overweight, those who have intractable pain [72], those with sleep challenges, those who cannot take medication, those at risk for falling, those at risk of poor adherence to regular exercise, and those who are depressed or anxious. These arthritis sub-groups groups may all be especially benefited [33,56,57,66,70,73,74], even if the exercises are only carried out for short periods, and the benefits are only perceived as benefits by the participants [75], such as increased ‘vitality’ [74] and ‘life quality’ [58].

## Figures and Tables

**Table 1:** Selected Problems Commonly Faced by People with Various forms of Arthritis.

• Persistent pain. • Joint inflammation and swelling. • Muscle weakness. • Poor endurance capacity. • Increased risk for falling. • Joint instability. • Limited joint flexibility and joint stiffness. • Limited mobility and function. • Obesity. • Poor bone health. • Poor posture. • Reduced balance capacity. • Stress, fatigue, sleep disturbances and lack of energy. • Depression and anxiety. • Lack of confidence in prevailing abilities to function, control pain. • Feelings of helplessness. • Reduced ability to work. • High levels of comorbid health conditions.

**Table 2:** Sample of Randomized Studies and Outcomes of Possible Relevance to Arthritis.

Author	Sample	Research and Intervention Strategy	Outcomes
Vera et al. [11]	43 health adults were randomized to an active or control group	Experimental subjects underwent daily Taoist Qigong training for 1 month	Statistically significant immune system benefits occurred in the experimental group
Chen et al. [17]	112 adults with knee osteoarthritis	External Qigong was applied by two practitioners and compared to sham intervention	Patients treated by healer 2 improved their pain score and function; overall function improved in both treatment groups
Lynch et al. [31]	100 participants were assigned to immediate or delayed treatment group	Qigong training (level 1 Chaoyi Fanhuan Qigong, CFQ), was given over 3 half-days, followed by weekly review/practice sessions for 8 weeks; participants were also asked to practice at home for 45–60 min each day	Significant benefits in pain, impact, sleep, physical and mental function occurred compared to wait-list/usual care control group at 8 weeks, with benefits extending beyond this period
Chan et al. [32]	150 chronic fatigue patients randomly assigned to experimental or control group	16 1.5 h sessions of Baduanjin Qigong exercise were held over 9 weeks	The number of Qigong lessons attended and amount of Qigong self-practice were significantly associated with sleep, fatigue, anxiety, and depressive symptom improvements Sleep improvements were maintained at 3 months
Tsang et al. [33]	50 chronically ill geriatric patients were allocated to an experimental or control group	Experimental subjects underwent 12 weeks of the Eight Section Brocades Qigong training; controls received standard care	Although not significant, the intervention group expressed improvements in physical health, activities of daily living, psychological health, social relationship, and general health
Gonzalez-lopez Arza et al. [34]	30 women 18–25 years of age were randomly assigned to experimental or control group	The Qigong group performed “exercises in 20 figures for health and long-life” (Wang Ziping) for 1 h twice per week, for 4 weeks. The control group undertook no exercise at all	Qigong improved balance in healthy women
Hwang et al. [35]	50 distressed individuals were randomized to an experimental or control group	A 4-week stress associated intervention using Qigong was conducted	Experimental subjects showed decreased stress and anxiety and improvements in life quality
Yang et al. [36]	43 elderly adults with chronic pain were randomized to an experimental or control group	Experimental subjects received 4 weeks of Qi therapy; controls received standard care	Compared with the control group, Qi-therapy participants showed improvements in mood and psychological variables over the 4 weeks. Pain and psychological benefits remained significantly improved after 2 weeks of follow-up
Haak et al. [37]	57 females with fibromyalgia were randomly assigned to experimental versus control group	Nine group sessions of Qigong for 7 weeks for a total of 11.5 h was implemented All subjects were encouraged to practice Qigong, with the support of a free instruction tape, twice a day at home External Qigong was given on 2 occasions The control group were given the same Qigong exercises, once the experimental group had completed their exercise regimen	Pain, psychological health, and distress levels were all improved for up to 4 months in the active participants

**Table 3:** Brief Summary of Key Findings reported by Lee et al. [40].

Variable	Experimental Group	Control Group
Quality of life	21.6 ± 16.8	9.8 ± 13.6
Walking time improvements	−1.6 ± 1.7	−0.2 ± 0.8
